# *Alu *distribution and mutation types of cancer genes

**DOI:** 10.1186/1471-2164-12-157

**Published:** 2011-03-23

**Authors:** Wensheng Zhang, Andrea Edwards, Wei Fan, Prescott Deininger, Kun Zhang

**Affiliations:** 1 Department of Computer Science, Xavier University of Louisiana, 1 Drexel Drive, New Orleans LA 70125, USA; 2 IBM T.J. Watson Research, 19 Skyline Drive, Hawthorne NY 10532, USA; 3 Tulane Cancer Center, Tulane School of Public Health and Tropical Medicine, New Orleans, Louisiana 70122, USA

## Abstract

**Background:**

*Alu *elements are the most abundant retrotransposable elements comprising ~11% of the human genome. Many studies have highlighted the role that *Alu *elements have in genetic instability and how their contribution to the assortment of mutagenic events can lead to cancer. As of yet, little has been done to quantitatively assess the association between *Alu *distribution and genes that are causally implicated in oncogenesis.

**Results:**

We have investigated the effect of various *Alu *densities on the mutation type based classifications of cancer genes. In order to establish the direct relationship between *Alu*s and the cancer genes of interest, genome wide *Alu*-related densities were measured using genes rather than the sliding windows of fixed length as the units. Several novel genomic features, such as the density of the adjacent *Alu *pairs and the number of *Alu*-Exon-*Alu *triplets, were developed in order to extend the investigation via the multivariate statistical analysis toward more advanced biological insight. In addition, we characterized the genome-wide intron *Alu *distribution with a mixture model that distinguished genes containing *Alu *elements from those with no *Alus*, and evaluated the gene-level effect of the 5'-TTAAAA motif associated with *Alu *insertion sites using a two-step regression analysis method.

**Conclusions:**

The study resulted in several novel findings worthy of further investigation. They include: (1) Recessive cancer genes (tumor suppressor genes) are enriched with *Alu *elements (p < 0.01) compared to dominant cancer genes (oncogenes) and the entire set of genes in the human genome; (2) *Alu*-related genomic features can be used to cluster cancer genes into biological meaningful groups; (3) The retention of exon *Alus *has been restricted in the human genome development, and an upper limit to the chromosome-level exon *Alu *densities is suggested by the distribution profile; (4) For the genes with at least one intron *Alu *repeat in individual chromosomes, the intron *Alu *densities can be well fitted by a Gamma distribution; (5) The effect of the 5'-TTAAAA motif on *Alu *densities varies across different chromosomes.

## Background

Classified as a Short Interspersed Element (SINE), *Alu *is the most abundant mobile element in the human genome [1,2]. A full-length *Alu *is approximately 300 nt in length and includes two tandem monomer units separated by a poly "A" stretch [3]. *Alu *elements are initially inserted fairly randomly throughout genome, with 5'-TTAAAA like motifs as preferred sites, and then accumulated over time in GC-rich regions through evolutionary selection [4-9]. *Alu *integration polymorphisms exist among individuals of the same population [10]. It has been a general recognition that *Alu *repeats play an important role in genome evolution, some cellular processes, DNA methylation, and transcriptional regulation [3,11-14].

In disease biology, the importance of *Alu *elements is further highlighted by the potential association with genetic instability, one of the principal hallmarks and causative factors in cancer [15,16]. *Alu*-mediated insertional mutagenesis and recombination have been reported in a few cancer genes [17-23]. Despite the rapid advances in *Alu *research, the rate and scope of the contribution of *Alu *to the origin and progression of human cancer is still poorly quantified to date [16]. As an endeavor to address this issue, a crucial task herein is to explore the association between genome-wide-spread *Alus *and mutated genes that are causally implicated in oncogenesis. However, the current knowledge about the known *Alu*-mediated cancer events is disproportional to that of the identified cancer genes. In a recently updated cancer gene database [24,25], 428 genes are verified to contribute to cancers; in contrast, only ten genes related to *Alu*-mediated insertional mutagenesis and recombination are cited in the most up-to-date literature [16]. Given *Alu*s are the most abundant retro-transposable elements in the human genome, the reported number of verified genes may account for only a small fraction of the potential cancer genes involved in *Alu*-mediated genetic instabilities. This fact greatly underscores the urgent need to conduct a genome wide association analysis of *Alus *and mutated cancer genes.

Numerous studies have been performed to investigate the distribution of *Alu *elements in the human genome. Some focus on how *Alu *repeats were integrated and spread in the human genome and the factors that may influence their distribution [2,4,9,26,27]. Others inspected the potential biological roles of these elements and their association with the genes of different functional categories in the specific chromosomes [28-30]. Because of *Alus*' potential contributions to genetic instability, in this paper, we attempted to investigate the effect of various *Alu *densities on the mutation feature based classifications of cancer genes. In order to establish more direct relationship between *Alu *repeats and the cancer genes of interest, "densities" were determined using genes as measurement units rather than the sliding windows of fixed length widely employed in practice [27,28,31]. Several novel genomic features, such as the density of adjacent *Alu *pairs and the number of *Alu*-Exon-*Alu *triplets, which may both contribute to *Alu/Alu *recombination rates that might influence gene function, were developed in order to extend the investigation via a clustering analysis toward more advanced biological insight. In addition, we characterized the *Alu *distribution with a mixture model that distinguished the genes with no *Alu *elements from those containing *Alus*. We also evaluated the gene-level effect of the predicted preferred integration site of *Alus*, 5'-TTAAAA, on the genome-wide *Alu *densities using a two-step regression analysis. These methods were especially proposed for analyzing the observed data where a large proportion of genes contain no *Alus *in their intron sequences. The study resulted in several important findings. In particular, we showed that recessive cancer genes are enriched with *Alu *elements compared to dominant cancer genes (oncogenes) and the entire gene set of the human genome.

## Results

### Chromosome-level *Alu *densities of different genome regions

**Figure 1:**
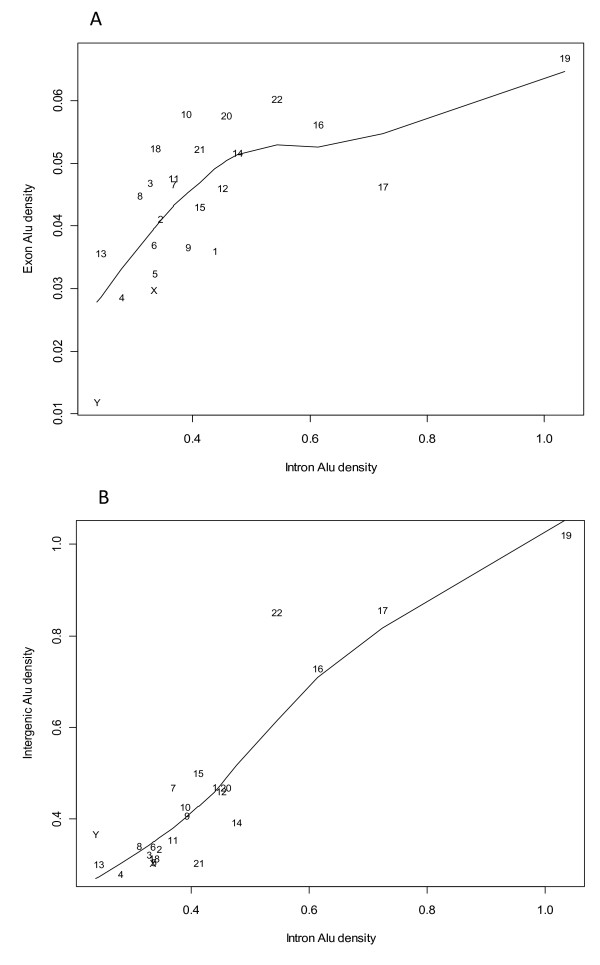
**The variability and association of chromosome-level *Alu *densities in different genomic regions**. The data points are labeled with chromosome IDs. The intron (exon or intergenic) *Alu *density is calculated by the number of intron (exon or intergenic) *Alus *divided by the corresponding sequence length measured in *Knt *(see the Methods section for details).

The location of *Alu *elements relative to genes is important in assessing their potential to contribute to gene disruption and genetic instability. Therefore, *Alu *densities in intron, exon and intergenic regions were calculated for individual chromosomes using the method described in the Methods section. The results were projected onto a two dimensional coordinate system (Figure 1A). Substantial variability exists across chromosomes. For example, chromosome-Y has the lowest intron and exon *Alu *densities, and chromosome-19 has the highest intron and exon *Alu *densities. The densities of intron and exon *Alu *elements in most of the chromosomes demonstrate a positive linear correlation if the three points corresponding to chromosome-Y, -17, and -19 are regarded as "outliers". The densities of exon *Alu *are approximately one order of magnitude lower than the *Alu *densities in introns. The intron *Alu *density of chromosome-19 is extremely high but its exon *Alu *density is comparable to those of chromosome-22 and -20. A possible explanation for this phenomenon is that exon *Alu *retention is under similar selective pressures on all chromosomes, while the intron *Alu *densities are controlled by different selective pressures. An upper limit to the chromosome-level exon *Alu *densities is suggested by the distribution profile. On the other hand, intergenic *Alu *densities and intron *Alu *densities are of the similar scale of magnitude. There is a strong positive linear relationship between the chromosome-level intergenic and intron *Alu *densities (Figure 1B). Based on this finding, we hypothesize that *Alu *elements in the intergenic and intron regions are under an analogous selection pressure or have not been selected in the evolution of the human genome. This, from another perspective, further highlights the distinct capability of exons from other genomic regions in retaining *Alu *elements.

### Distributional characterization of gene-level intron *Alu *density

**Figure 2:**
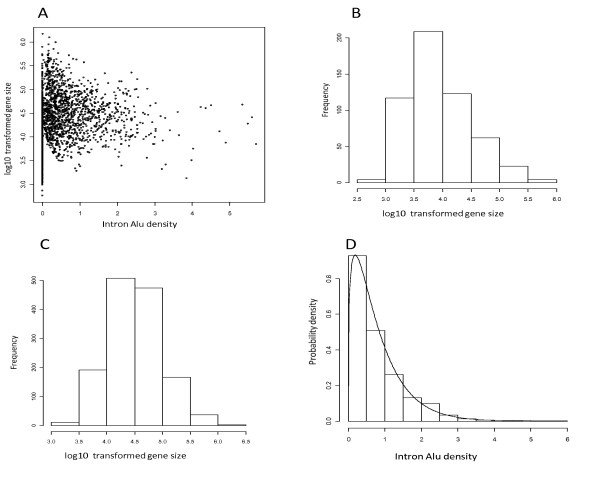
**The distributional characterization of intron *Alu *density in chromosome-1**. **A: **the scatter plot of intron *Alu *densities versus log10 transformed lengths of gene sequences. **B: **The distribution of the lengths of the multi-exon genes without any *Alu *elements in the intron sequence(s). **C: **The distribution of the lengths of the multi-exon genes with at least one *Alu *element in the intron sequence(s). **D: **The distribution of intron *Alu *densities of the multi-exon genes with at least one *Alu *in the intron sequence(s). The curve represents a gamma density function with 0.615 and 1.313 as the shape and scale parameters, respectively.

As discussed above, the number of fixed *Alu*s in exon regions of genes is quite small compared to that in intron regions. Therefore, we focused on characterizing the distribution of gene-level *Alu *density in intron regions. In the UCSC gene annotation released in 2006, approximately 12% of the human genes (21461) are of single exon and have no intron sequences in the transcript(s), thus those single-exon genes were excluded. The 18856 multi-exon genes are kept for further analysis. As an illustration, with the log10 transformed gene size (sequence length) as X-axis and the intron *Alu *density as Y-axis, we projected 1928 multi-exon genes in chromosome-1 onto a two-dimensional coordinate system (Figure 2A). Regardless of the gene size, 28.1% of these multi-exon genes in chromosome-1 contain no *Alu *elements in their intron regions. We selected those genes and generated a histogram of their log10 transformed sizes (Figure 2B). Similarly, we generated the histogram for the multi-exon genes with at least one intron *Alu* (Figure 2C). Both profiles are largely in the shape of a normal distribution. Compared to Figure 2B, the center of Figure 2C shifts 0.5 units in logarithmic scale to the right, indicating that the geometric mean of the multi-exon genes with intron *Alus *is approximately three times of that of the multi-exon genes without any intron *Alus*. (This ratio also holds for the whole genome). Figure 2D presents the histogram of the intron *Alu *density of the 1386 genes with at least one *Alu *in their intron region(s). The curve represents a fitted Gamma probability density function with 0.615 and 1.313 as the shape and scale parameters, respectively. The rationale and formulation of the mixture model analysis of gene-level intron *Alu *density are discussed below. It should be noted that, gene-level intron *Alu *densities in the other 23 chromosomes also carry the similar Gamma distribution characteristic (see Table 1 for the estimated model parameters).

**Table 1:** The mixture model analysis of intron *Alu *density

**Chr**.	N	r	θ	κ
chr1	1928	0.281	1.303	0.615
chr2	1212	0.271	1.498	0.441
chr3	1036	0.248	1.398	0.459
chr4	728	0.26	1.573	0.31
chr5	837	0.238	1.406	0.42
chr6	952	0.279	1.369	0.47
chr7	893	0.308	1.157	0.735
chr8	667	0.315	1.553	0.403
chr9	734	0.342	1.656	0.411
chr10	749	0.288	1.509	0.457
chr11	1072	0.328	1.349	0.528
chr12	984	0.269	1.262	0.71
chr13	325	0.295	1.881	0.246
chr14	562	0.285	1.667	0.475
chr15	580	0.284	1.367	0.59
chr16	811	0.337	1.74	0.617
chr17	1106	0.344	1.782	0.617
chr18	262	0.214	1.694	0.329
chr19	1342	0.286	1.586	1.002
chr20	537	0.307	1.363	0.582
chr21	203	0.241	1.758	0.355
chr22	452	0.352	1.762	0.595
chrX	778	0.356	1.241	0.471
chrY	93	0.688	2.098	0.217

**N: **the number of genes with multiple exons in the NCBI reference sequences. **r: **the proportion of the multi-exon genes without any *Alus*. **θ: **the shape parameter of the fitted Gamma distribution. **κ: **the scale parameter of the fitted Gamma distribution.

### Mixture model analysis of gene-level intron *Alu *density for multi-exon genes

Because of the significant number of genes without any *Alu *elements, the descriptive statistics, such as mean and standard deviation, are not sufficient to characterize the gene-level *Alu *distributions for the genes in the individual chromosome. Furthermore, the separation of the presence or absence of *Alu elements *from the continuous density measures may be important in the investigation of the mechanism underlying the *Alu *insertion and retention. Based on these considerations, we characterized the gene-level intron *Alu *density distribution with a mixture model that consists of a Bernoulli probability mass function and a Gamma probability density function. The summary statistics included , , and .  is the proportion of the genes without any intron *Alus*.  and  are the shape and scale parameters of the Gamma function that describes the empirical distribution of *Alu *densities of the genes with at least one intron *Alu *element. The details of the model are presented in the Methods section.

**Figure 3:**
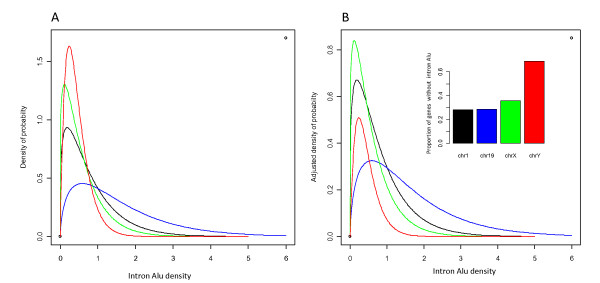
**The characterization of intron *Alu *densities of multi-exon genes in chromosomes-1, -19, -X and -Y with a mixture model**. **A: **the curves represent the probability density functions given the intron *Alu *density larger than zero. **B: **the probability densities are adjusted according to the proportion of the genes without *Alus*. For each chromosome, the sum of its proportion of genes without intron *Alus *and the area under the Gamma curve equals to one.

**Figure 4:**
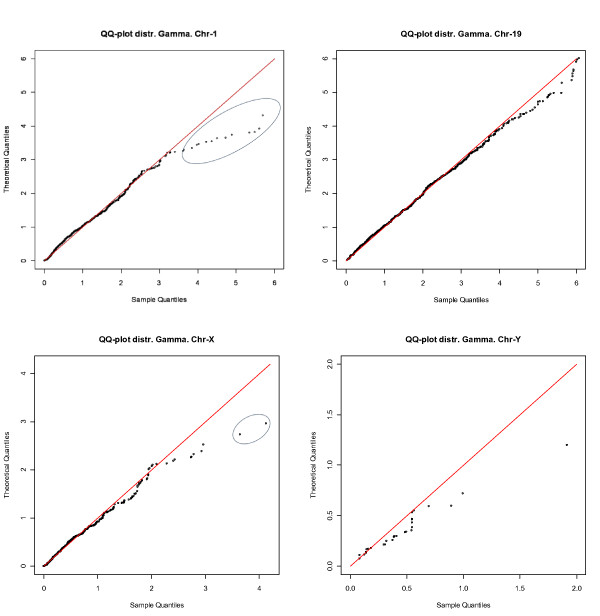
**The Q-Q plots for fitting a Gamma distribution to the gene-level intron *Alu *densities in chromosomes-1, -19, -X and -Y**. The dots represent genes. The genes in the ovals on the plots for chromosome -1 and -X are regarded as outliers and further analyzed in Additional file 2.

Table 1 lists the estimated model parameters for all 24 chromosomes. Using those parameters, we can clearly visualize the intron *Alu *distribution of each chromosome and demonstrate the differences among them. In Figure 3A, the curves represent the theoretically calculated distributions of intron *Alu *densities of the genes containing at least one *Alu *for chromosomes -1, -19, -X and -Y. In Figure 3B, the probability density is adjusted based on the proportion of the genes without any intron *Alus *such that, for each chromosome, the sum of  and the area under the curve is equal to one. Figure 4 shows the Q-Q plots of the four chromosomes characterized in Figure 3. Those plots were generated to evaluate how well the estimated Gamma distributions described the observed intron *Alu *densities. Each dot on the plots represents a gene and the rightmost genes have the highest intron *Alu *density. The top left plot corresponds to the Gamma curve of chromosome-1. It is evident that, in general, the estimated Gamma curve fits the observed data except for a few outliers at the tail. The Q-Q plot for chromosome-19, the *Alu*-richest chromosome, is almost perfect in that nearly all points lie close to the diagonal line, indicating a good fit. Similar to chromosome-1, the plots for the two sex chromosomes have several outliers at the tails. The highest degree of deviation can be observed from the plot of chromosome-Y, which is the least *Alu *dense chromosome. After examining the plots for the rest of the chromosomes (Additional file 1), we can draw the general conclusion that the proposed model fit the *Alu*-rich chromosomes better than the *Alu*-poor chromosomes. For each chromosome, the overall good fitting of a right-skewed Gamma distribution to the intron *Alu *density of the *Alu*-containing genes indicated that the bulk of genes have relatively low *Alu *density with fewer genes retaining very high *Alu *densities.

The information of 47 genes on chromosomes 1,3,6,13,21 and X that apparently deviate from the diagonal lines in Q-Q plots is summarized in Additional file 2. We found that these genes cluster in a few chromosome regions and approximately one third of them have high exon *Alu *density which is not taken into account in the modeling. We did not note any over-represented functional groups of these genes, however, and believe that the high *Alu *density of these genes may be mainly influenced by their chromosomal region rather than their function.

### Distributions of *Alu*s and 5'-TTAAAA motifs

It has been widely recognized that the integration of *Alu *elements is initiated with its endonuclease-dependent cleavage at the 5'-TTAAAA hexanucleotide, and the variants derived by a single base substitution, particularly from A to G and T to C [4,8]. A recent publication further analyzed the effects of genomic features, including the density of 5'-TTAAAA, on the *Alu *density using a multiple regression method [9]. The authors divided the entire human genome into around 2400 bins with each of 1 M bases long, and measured motif and *Alu *densities on these sequences of fixed length. In our study, the densities were determined using genes as the units, thus our data structure and representation was quite different from that in [9]. Considering the scarcity of the retained *Alu *elements in the exon regions, we focused on the analysis of the motif in introns relative to *Alu *densities. The substantial level of genes without any *Alus *makes the method employed in [9] not applicable to the statistical analysis conducted here.

**Figure 5:**
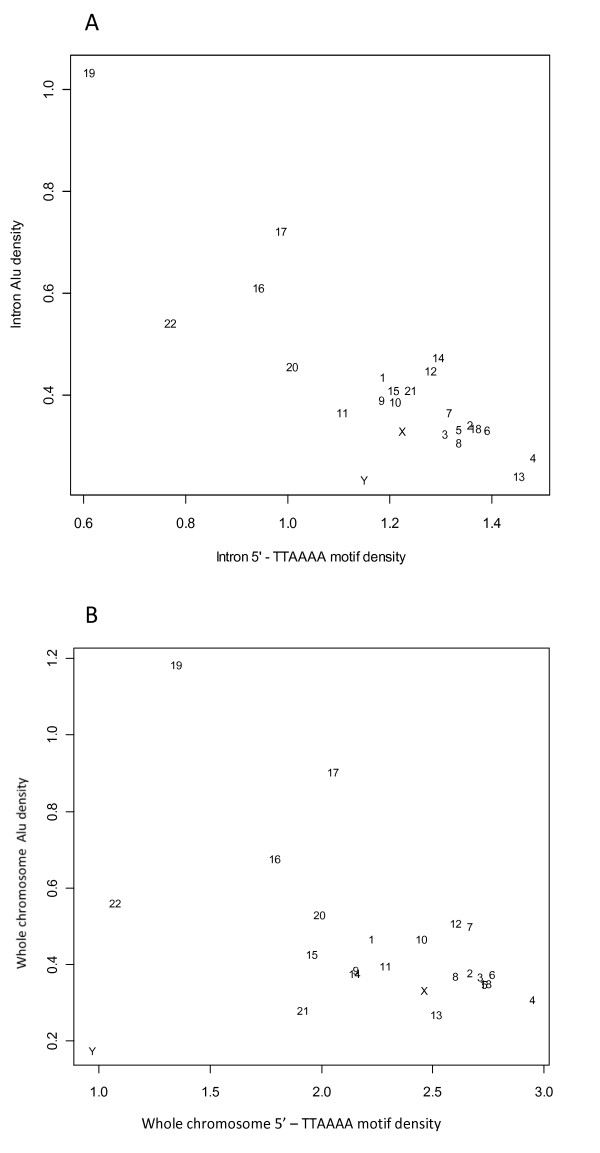
**The correlation between chromosome-level *Alu *density and 5' - TTAAAA motif density in introns and in the individual chromosome**. The data points are labeled with chromosome IDs.

Figure 5 presents the chromosome-level *Alu *and 5'-TTAAAA motif densities in introns and in each individual chromosome, respectively. A striking negative correlation between *Alu *density and 5'-TTAAAA motif density can be observed from both plots regardless of the measurements performed in introns or in the whole chromosome. These data suggest that either the density of potential insertion sites is not rate limiting, or that the relative density of *Alu *elements is governed more by selection than by initial insertion density.

#### Two-step regression analysis of the gene-level effect of 5'-TTAAAA motif density on Alu density

**Figure 6:**
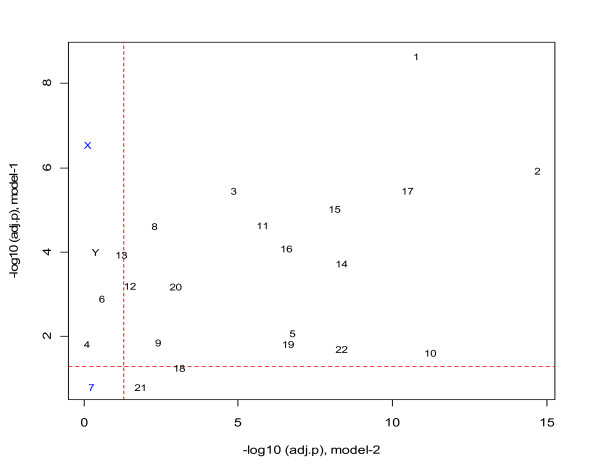
**The visualization of the gene-level effects of 5'-TTAAAA intron motif density on intron *Alu *density obtained from the proposed two step regression method**. The data points are labeled with chromosome IDs. The X- and Y- coordinates are the negatives of the log10 transformed p-values (adjusted with BH method) from the two models, respectively. The dashed red lines correspond to 0.05 in the scale of adjusted p-values. Model-1 evaluates the effect of the motif density on the presence or absence of *Alus *in the introns of the genes. Model-2 evaluates the effect of the motif density on the intron *Alu *density of the genes with at least one *Alu *repeat in their intron regions. All the regression coefficients β are positive in the model-1 and the coefficients β* are positive in model-2 except for chromosomes 7 and X (marked with blue).

We conducted a more refined study to analyze the effect of 5'-TTAAAA motif density on intron *Alu *density at the gene level. Figure 6 shows the results obtained from the proposed two-step regression method (see the Methods section for details). The data points are labeled with chromosome IDs. The X- and Y- coordinates are the negatives of the log10 transformed FDR-adjusted p-values [32] from the two models, respectively. The dashed red lines correspond to 0.05 in the scale of adjusted p-values. Model-1 evaluates the effect of the motif density on the presence or absence of *Alus *in the gene introns. Model-2 evaluates the effect of the motif density on the intron *Alu *density of the genes with at least one *Alu *repeat in their intron regions. As shown in Figure 6, the influence of motif density on the intron *Alu *density is chromosome-specific. For chromosome-7, the effect is not significant as demonstrated by both models. For chromosomes -4, -6, -13, -X, and -Y, the effect is significant only in Model-1. For chromosomes -18 and -21, the effect is significant only in Model-2. The rest of the chromosomes have the adjusted p-values less than 0.05 as reflected by both models. The most significant cases are detected in chromosome-1 where the adjusted p-values are less than 1 × 10^-8 ^and 1 × 10^-10 ^in the two models, respectively. For chromosome-19 which has the highest genome-wide *Alu *density, the effect is marginally significant as measured by Model-1. The chromosome-wide gene-level positive association between *Alus *and the motif are suggested by the facts that all the regression coefficients β are positive in the model-1 and the coefficients β* are positive in model-2 except for chromosomes -7 and X.

### Correlations of *Alu *density with the genetic classifications of cancer genes

We analyzed the effect of *Alu *density on the classification of 428 cancer genes collected in the recently updated (May 2010) COSMIC database [25]. The three classifications evaluated are:

(1) Dominant or recessive mutations;

(2) Somatic or germline mutations;

(3) Translocation or non-translocation mutations.

We filtered out 77 genes that either contained only a single exon or were not included in the UCSC 2006 annotation, and kept the remaining 351 genes for further analysis. In addition, 30 genes with the mutation found in both cell types were excluded from the comparison of somatic and germline mutations, and 2 genes labeled with "Rec?" in the database were excluded from dominant and recessive mutation comparison.

**Figure 7:**
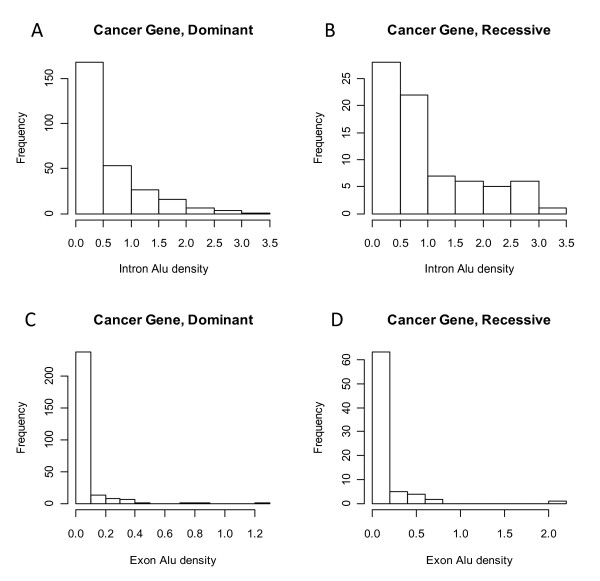
**The frequency distributions of intron and exon *Alu *densities of the cancer genes with dominant and recessive mutations**.

Figure 7 shows the frequency distributions of intron *Alu *densities (**A **and **B**) and exon *Alu *densities (**C **and **D**) of the cancer genes with dominant mutations (n = 274) and recessive mutations (n = 75), respectively. The class of recessive genes, also known as tumor repressor genes, has higher *Alu *densities. In particular, approximately 63% of recessive genes, compared to 19% of dominant genes, have intron *Alu *densities greater than 0.5. Statistical analysis using a logistic regression model (see the Methods section) further demonstrated that, on the dominant/recessive mutation types based classification, the effect of intron *Alu *density is extremely significant (p <.001) and the effect of exon *Alu *density is marginally significant (p < .05). The comparison of the genes with the mutations found in somatic cells (n = 285) or germline cells (n = 36) is shown in Additional file 3. The class of genes with germline mutations demonstrated higher *Alu *densities in that approximately 64% of germline genes, in contrast to 40% of somatic line genes, have over 0.5 intron *Alu *densities. On the germline/somatic classification, the effect of intron *Alu *density is extremely significant (p <.001) and the effect of exon *Alu *density is marginally significant (p < .05). In the last comparison (Additional file 4), only intron *Alu *density demonstrates significant effect (p = 0.013) on the translocation/non-translocation mutation types based classification. The class of genes with translocation mutations (n = 231) has lower *Alu *density than the genes with non-translocation mutations (n = 120). The corresponding proportions of genes with intron *Alu *density over 0.5 in the translocation/non-translocation mutations are 44% and 57%, respectively.

### Enrichment analysis of *Alu *elements and mutation types in cancer genes

**Figure 8:**
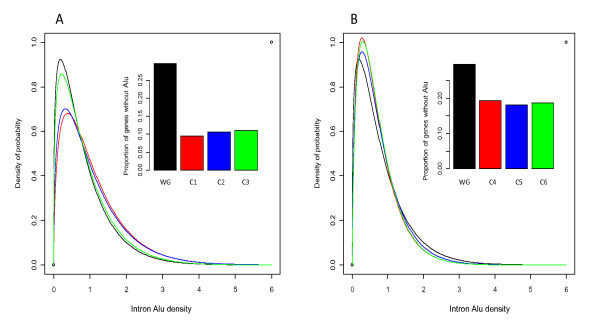
**The comparison of estimated distributions of intron *Alu *densities in different cancer gene classes**. The proposed mixture model method was used in this analysis. The curves represent the approximated Gamma distributions of *Alu *density for the genes with at least one intron *Alu*. The column charts represent the proportions of genes without any intron *Alus*. **WG: **the entire set of multiple-exon genes in human genome. **C1: **the cancer genes with recessive mutations. **C2: **the cancer genes with mutations found in germline. **C3: **the cancer genes with non-translocation mutations. **C4: **the cancer genes with dominant mutations. **C5: **the cancer genes with somatic mutations. **C6: **the cancer genes with translocation mutations. See **Figure 7**, Additional files 3 and 4 for the empirical distributions of *Alu *densities for the above six classes C1-C6.

*Alu *mediated mutagenesis events have been widely reported in the literature and most of the studied cancer genes contain a considerable number of *Alu *elements in their sequences. In order to highlight the differences among the studied cancer gene classes, we used the mixture model as discussed above to visualize the observed results (Figure 8A). The distributions of the intron *Alu *densities of the three cancer gene classes, i.e. recessive mutation (C1, n = 75), germline mutation (C2, n = 36) and non-translocation mutation (C3, n = 120) differ from the profile of the entire multiple-exon genes (N = 18856) in the human genome in both the proportion of the genes without *Alus *(as shown by the column charts) and the genes with at least one *Alu *element (described by the Gamma curves). A logistic regression analysis shows that the association between these gene classes and *Alu *intron density are significant (p < 0.01). This statistical model was established with *z *∈{1,0} (indicating whether or not a gene belongs to a mutation class) as the dependent variable, and the intron *Alu *density and log10 transformed gene size as the explainable variables.

Figure 8B presents the distributions of the intron *Alu *densities of another three cancer gene classes, i.e. dominant mutation (C4, n = 274), somatic mutation and (C5, n = 285) and translocation mutation (C6, n = 231). As demonstrated by the Gamma curves describing the genes with at least one *Alu *element, the distributions of these three classes are similar to the profile of the entire multiple-exon genes in human genome. Moreover, the logistic regression analysis shows that the association between these gene classes and *Alu *intron density is not significant (p > 0.05).

### Clustering analysis of cancer genes based on *Alu*-related genomic features

**Figure 9:**
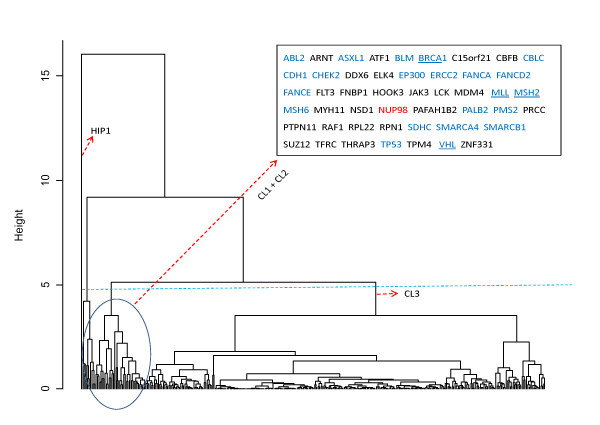
**The clustering analysis of cancer genes using *Alu*-related genome features and GC content**. In the text box, 22 recessive cancer genes (tumor suppressor genes) are highlighted in blue. The BRCA1, MLL, MSH2 and VHL genes (marked with underlines) are among the seven cancer genes in which *Alu *mediated recombination events have been reported [16]. NUP98 (marked with red) was classified as dominant mutation in the COSMIC database but located at a cancer suppressor gene region [33]. The gene that appears as a scalar in the dendrogram is the Huntingtin interacting protein 1 gene (HIP1).

In order to further investigate the association between *Alu *density and mutagenesis by using other information besides intron *Alu *density, we conducted a clustering analysis on the 351 cancer genes as mentioned above. Figure 9 shows the dendrogram generated by applying the agglomerative hierarchical clustering algorithm to the gene set described by four *Alu*-related genomic features and GC content (see the Methods section for details). Based on the biological insight that can be derived from all possible groups, we chose to cut the tree at the height of 4.5 heuristically, and aggregated the 351 genes into a scalar (HIP1) and three clusters. The two smaller clusters (CL1 and CL2) contain 48 genes in total. Among them, the recessive cancer genes, also known as tumor suppressor genes, account for 45.8%. This proportion is 2.5 times of the corresponding ratio (53/302 = 17.6%) in the third cluster (CL3). Statistical analysis using Fisher's exact test demonstrates that this difference is not due to chance (p = 3.8 × 10^-5^). Furthermore, gene NUP98, classified as dominant mutation in the COSMIC database but located at a tumor suppressor gene region [33], is also included in this set of 48 genes. Among the seven genes with numerous *Alu *mediated recombination events reported [16], four genes (BRCA1, MLL, MSH2 and VHL) are contained in CL1 and CL2. Another two (MYB and MLH1) are grouped into CL3 and the remaining one (hCAD) is not among the COSMIC gene list. This suggests that the clustering analysis may provide a promising gene list for further investigation of more *Alu *mediated recombination events. In order to reveal the relative importance of the used genomic features in the clustering, we sorted the 351 genes in terms of the intron *Alu *density and visualized the results using five bar-plots (Additional file 5). It is evident that the genes within CL1 and CL2 have much higher intron *Alu *density (as well as intron *Alu *pairs) than the genes in CL3. HIP1 is distinguished from other genes due to its large number of *Alu*-exon - *Alu *triplets. On the other hand, not much differential information can be inferred from the rather comparable GC content of those cancer genes.

## Discussion

We entered this study with the biological observation that *Alu *elements contribute to genetic instability by insertional mutagenesis, and then deletions/duplications through *Alu*/*Alu *non-allelic homologous recombination [16]. Based on this perception, we hypothesized that local *Alu *integration rate or density may have unequal significance to the mutations of different functional classes or biological groups of cancer genes that are usually predominated by one or multiple special genetic mechanisms. While our primary effort focused on bioinformatically testing this hypothesis, we also modeled the genome-wide, different-levels of relationships between the 5'-TTAAAA density and *Alu *distribution. The latter study is relevant in that the *Alu *cleavage related to this motif by the L1 endonuclease is not only a key first step in *Alu *integration [4], but may also help lead to DNA double-strand breaks that could contribute to *Alu*/*Alu *non-allelic homologous recombination events and genetic instability in motif-rich genes. With respect to the methodology, besides characterizing the genome-wide intron *Alu *density using a mixture model and analyzing the effects of 5'-TTAAAA on the *Alu *integration through a two-step regression approach, we devised the use of *Alu *pairs and *Alu*-exon-*Alu *triplets as additional means of targeting those sets of *Alus *that are most likely to contribute to genetic instability within genes. Moreover, the association between these *Alu*-related genomic features and the different types of cancer genes was studied using a logistic regression model and an un-supervised learning method. Because the impact of this genetic instability on genes is the most critical aspect to human disease, we chose to carry out all analyses on individual genes as the key unit, with a particular focus on cancer genes where genetic instability is known to be a major contributor to the disease. Through the rigorous statistical analysis, we observed the hypothesized association between the *Alu *density and mutation type of cancer genes. In the following, we focus on some results that require further discussion.

### Exon *Alu *density

Exonized *Alus *(insertion within an intron that led to exon creation) and exonic *Alus *(insertion into existing exons) were summed together in the calculation. It is reasonable to assume that *Alu *elements initially integrating into exon sequences have led to strong negative selective pressures limiting their accumulation [11]. The overall positive linear correlation between the chromosome-level exon *Alu *densities as shown in Figure 1A suggests the approximately equal chance (across chromosomes) of the retention of the initially inserted *Alus *in exon regions. On the other hand, the observed upper limit to the chromosome-level exon *Alu *density may indicate that the fixation of *Alu *elements in the coding regions of genes is further determined by the biological tolerance. The hypothesis of the negative selection of *Alus *in exon regions is also supported by the simple positive linear relationship of *Alu *densities in intron and integenic regions (Figure 1B).

### Distributional characterization of *Alus*

If a gene has no *Alu *repeats in its intron region(s), the intron *Alu *density of this gene will be zero. As illustrated by genes on chromosome-1 (Figure 2A), the sizes of genes without *Alus *nearly span all possible lengths. Due to the substantial existence (N = 5578) of multi-exon genes of such kind in human genome, we characterized the distribution of the intron *Alu *density with a mixture model that distinguished genes without *Alu *elements from those containing *Alus*. The main advantages of this method include: (1) it provides a more insightful summary of *Alu *distribution than simple statistics such as means and standard deviations for the observed data with complex structures [34]; (2) the separation of *Alu *presence or absence from the continuous density measures may be important in the investigation of the mechanism underlying the *Alu *insertion and retention. It is worth noting that we can replace the Gamma distribution with another member of exponent family, i.e. Weibul distribution. We prefer the Gamma distribution because the first and second moments (expectation and variance), which may be of interest to some researchers, can be directly calculated from the model parameters using two simple formulas, i.e. *E*(*X *) = *θκ *and var(*X *) = *θκ*^2 ^[34]. Power law-like distributions, such as the Gamma distribution, have been widely used in describing genomic features [31,35] and empirically found to fit many basic population models [36]. Our model suggests that genes containing *Alu *elements have an excellent, though not perfect, fit to a gamma distribution (Figure 4), indicating a similarity between *Alu *distribution in genes and natural population variation. Deviations from a perfect fit might suggest that some genes either have a less than normal selection against the presence of *Alu *elements or have either a sequence preference for the element that leads to unusually high *Alu *buildup.

For multi-exon genes without intron *Alus*, an interesting question worth further study is why they lack this type of repeats. Is it because those genes are located in *Alu*-poor regions or their structures or (and) functions don't tolerate *Alus *after the initial insertion? A functional enrichment analysis using DAVID tool [37] showed that 64 level-5 (most specific) GO terms, of which 77% belong to the general category of biological process, are over-represented by those genes with the FDR adjusted p-values less than 0.01 (Additional file 6). The result is different from a recent study that stated that "no evidence for selective loss of these elements in any function class." [38]. We are conducting a more comprehensive investigation on this issue.

### Intron *Alu *density and 5'-TTAAAA motif density

As specified by [4], 5'-TTAAAA is the most abundant hexanucleotide signal for the primary integration of *Alus*. A recent publication further reported that such motif(s) contributed to 6.1% - 26.7% of the variation in *Alu *density of genome sequences of fixed length, depending on the subtypes and the genome regions related to the evolution divergence of human, chimpanzee and orangutan [9]. Our study shows that the gene-level effect of the motif density on *Alu *density varied across chromosomes substantially in terms of statistical significance level. However, except for chromosome-7, all other chromosomes have the adjusted p-values less than 0.05 at least in one of the two proposed statistical models that evaluated the motif effects from different aspects. The (pseudo) contribution rates to the total variability are lower than 11%, in general. One exception occurred in chromosome-Y where the *Alu *distribution holds special importance in studying the evolution of genome. For this chromosome, the pseudo contribution rate is as high as 33% in Model-1 where the binary dependent variable indicates if a gene contains at least one *Alu *in its intron region(s). A possible explanation for this exception is that the redistribution of *Alus *on this sex chromosome was relatively delayed due to the lack of recombination between chromosome pairs [27]; therefore, the initial association between the motif and *Alus *has been largely retained. Here, one may be puzzled that the adjusted p-value calculated from Model-2 (indicating the gene-level effect of 5'-TTAAAA motif on intron *Alu *density) for chromosome Y is larger than 0.05. While the reason is still not clear, we tend to attribute the inconsistence to the fact that, in our dataset, chromosome-Y has only 29 genes containing *Alus *and thus the Model-2 lacks power to detect the *Alu*-motif association for this chromosome

Surprisingly, we also found a fairly strong negative correlation between the density of this motif and *Alu *element density on a chromosome basis. This suggests that either there is little correlation of the density of L1 endonuclease cleavage sites and the final density of *Alu *elements, or that the post-insertional selection process has largely led to the dissociation of these *Alu *density from its initial insertion density. Evolutionary analysis of older vs. younger *Alu *elements [27], as well as de novo *Alu *insertions in tissue culture [39-43], strongly support the latter hypothesis. It has been argued that *Alu *elements are selectively retained in genes of specific classes [38]. We would argue that *Alus *insert initially fairly randomly and there is variable selective loss by genes, but the most significant factor is likely to be that genes that are particularly sensitive to the genetic instability caused by *Alu *elements through recombination may retain the *Alus *because those elements cannot be lost through recombination. On the other hand, it is reasonable to assume that the association between the 5'-TTAAAA motif and *Alu *elements in these genes have been retained, leading to the positive correlation as shown at the gene-level for individual chromosomes.

### *Alu*-enriched cancer genes

In the Result section, we firstly showed that intron *Alu *density has a significant impact on several binary classifications of cancer genes established on the mutation types provided by COSMIC. Then, by comparing those gene categories with the entire gene set in the human genome, we found that in each classification pair, only one class is enriched with *Alus*. The *Alu*-enriched categories (p < 0.01) include recessive mutation class (C1), germline mutation class (C2), and non-translocation class (C3). However, because about 90% of genes in germline mutation class are also recessive, we actually have two relatively independent *Alu*-enriched classes, i.e. C1 and C3. Furthermore, because the classification based on germinal and somatic mutations can be well explained by the classification in terms of dominant and recessive mutations [24], here we concentrate our analysis on the implications behind the associations between the *Alu *density and the dominant and recessive mutations.

Our findings relative to these cancer-causing genes fall largely into the distribution expected for genes influenced by genetic instability. For instance, oncogenes or dominant cancer genes are often subject to smaller mutations than those caused by *Alu *insertion or recombination. *Alu *elements more typically disrupt gene expression or cause loss of part of a gene, and this is more typical of the mode of cancer generation by the recessive cancer genes or tumor suppressors. The high density of *Alu *elements in tumor suppressor genes would then cause increased risk of *Alu*/*Alu *recombination leading to cancer. Although it is somewhat counterintuitive to think that *Alu *elements would build up in genes that have such potential sensitivity to the genetic instability that they would cause, one might explain it by considering that once an *Alu *is present in the gene, it is difficult to allow it to be removed by a recombination-based process. At first glance, the finding of a negative correlation between *Alu *elements and translocation mutations might seem surprising. *Alu *elements have been shown to be capable of undergoing non-allelic homologous recombination between chromosomes [44] that could contribute to such translocations. However, the vast majority of chromosomal translocations, particularly those associated with cancer, have been found to be formed by non-homologous end-joining, a process that would not be influenced by *Alu *elements.

As discussed above, the main message conveyed by this paper is that cancer genes have different *Alu *insertion (or retention) rates according to the genes' type of mutation. This is a rigorous conclusion in the sense that, prior to reaching it, we explored the potential influence of other factors on the statistical significance of the discovered association. That is, in a preliminary study, we analyzed the correlation of various genomic features, such as the gene length, exon number, exon/intron length ratio and the density of the recombination hot spots-related motif CCTCCCT [45], with the studied classifications of cancer genes. The results showed that the only significant factor is the gene length (p < 0.05), therefore we included this feature in the final statistical models as described in the Method and Result sections. Another relevant concern is that the adopted classification of cancer genes may be biased because, in the search for tumor suppressor genes, special effort has been made in genomic regions undergoing frequent losses. In this regard, we retrieved the middle coordinates of 129 documented fragile sites (FS) in human genome assembly 36 [46,47]. Based on the coordinates and the genes' locations, we calculated the distance between each studied cancer gene to its nearest FS. The subsequent statistical analysis showed that the distances did not significantly differentiate among the different gene classes (p > 0.1). This indicates that our conclusion on the association between the *Alu *density and mutation types of cancer genes is still valid even when the genes' physical locations relative to the unstable genome regions are considered. The mechanism behind the fairly high *Alu *density in recessive cancer genes is still unclear. One possible explanation is that, except for a few "dominant negative" cases such as those in TP53 [48], an *Alu *mediated mutation including insertion and recombination events may less likely change the protein-coding sequences (exons) such that the chances leading to novel proteins, especially those with lethal effects, may be relatively low, thus only in the homozygous status can the mutated allele produce deleterious effect.

### Knowledge discovery based on *Alu*-related genomic features

It has been suggested that, besides the intron *Alu *density, the existence or absence of exon *Alus*, the relative location among *Alus*, the proximity of *Alus *to exons, and the GC content in the gene sequences are also important factors in the *Alu *mediated mutagenesis events [16]. In this study, the clustering analysis of cancer genes using these genomic features demonstrated a clear hierarchical structure as shown in Figure 9. The significant association between the clusters and the genetic classification of cancer genes is identified from the advanced statistical analysis. This result suggests that the potential merits of using these features to predict recessive cancer genes and *Alu *mediated recombination events. Because the majority of currently documented cancer genes are dominant, it is reasonable to assume that many recessive cancer genes remain undiscovered. Volina *et al *recently proposed an approach to identify the genome-wide recessive cancer genes by combining the contributions of the different types of genetic alterations to loss of functions [49]. The method was promising but without remarkable sensitivity. It is possible to extend this study by integrating the measures used in [49], i.e. amino-acid substitutions, frame-shifts and gene deletions, with the *Alu*-related genomic features for a more insightful exploration.

## Methods

### Data sources

Chromosome DNA sequences and gene annotation information (including the official symbols, orientations, and coordinates of NCBI reference gene sequences and exons) were retrieved from UCSC tables for NCBI36/hg18. Single exon genes (without any introns in the reference sequence) were excluded from further analysis because they lack information of *Alu *integration. The coordinates of intron *Alus*, exonic *Alus *and exonized *Alus *were extracted from the AluGene database [50,51], which is established by applying the RepeatMasker software to hg18 [52]. The coordinates of 5'-TTAAAA motifs were identified by using the R package "Biostrings" on hg18. The information of cancer genes was obtained from Catalogue of Somatic Mutations in Cancer (COSMIC) [25].

### Density and other related calculations

#### Gene level densities

For each reference gene, the *Alu *density in its intron or exon region (or region clusters) was determined by the number of intron or exon *Alus *and the corresponding adjusted sequence length with the nucleotides contained in the *Alus *being excluded from the calculation of the sequence length. More specifically, the number of *Alus *in a NCBI gene reference sequence (Nt) and the number of *Alus *in the exon region(s) (Ne) were respectively counted. Exon *Alus *included exonized *Alus *(insertion within an intron that led to exon creation) and exonic *Alus *(insertion into existing exons). The number of *Alu*s in the intron region(s) (Ni) was calculated by subtracting Ne from Nt. The adjusted intron sequence length (Si) was calculated by subtracting the total length of exon(s) and intron *Alu*(s) from the gene sequence length. The adjusted exon sequence length (Se) was calculated by subtracting the total length of exon *Alu*(s) from the total length of exon(s). Intron *Alu *density (Di) and exon *Alu *density (De) were computed by the following formulas. All sequence lengths, such as Si and Se, were measured in terms of *kilo nucleotides*, abbreviated as *Knt*.(1)

For each reference gene, the 5'-TTAAAA motif density in the region (or region cluster) of intron or exon was determined by the number of the motifs and the corresponding adjusted sequence length with the nucleotides in the *Alus *excluded from the sequence length calculation. The details were similar to the computation of *Alu *densities as mentioned above.

Finally, the determined *Alu *(motif) densities were adapted to the gene names present in the UCSC genome browser. For a gene annotated with multiple reference sequences (transcripts) in the same chromosome and strand, the *Alu *(motif) densities were obtained by calculating the mean.

#### Gene level intron Alu-pair density

An *Alu*-pair forms when the distance between two adjacent intron *Alu *elements is less than 300 bases. The density was calculated by the number of *Alu*-pairs divided by the adjusted intron sequence length. The number of *Alu*-pairs in each reference (gene) sequence was counted individually. For a gene annotated with multiple reference sequences, we calculated the average value.

#### Gene level number of Alu-exon-Alu triplets

An *Alu*-exon-*Alu *triplet was defined as an exon flanked by two *Alus *with the distance of each interval (*Alu*-exon or exon-*Alu*) less than 300 bases. The number of such triplets in each reference (gene) sequence was counted individually. For a gene annotated with multiple reference sequences, we calculated the average triplet number.

#### Gene level CG content

To unbiasedly estimate the CG content that can reflect the genomic environment for *Alu *integration, we excluded the nucleotides contained in the *Alu *elements from the calculation. This is different from the common practice employed in the literature.

#### Chromosome level densities

For each chromosome, its intron (exon, intergentic) *Alu *(motif) density was calculated by dividing the total number of intron (exon, intergenic) *Alu*s (motifs) by the adjusted total intron (exon, intergenic) sequence length. Same as the gene-level density calculation described above, all sequence lengths were measured in terms of *Knt*, and "adjusted" means that *Alu *sequences were excluded from the sequence length calculation. An intergenic sequence was approximately determined as the genome section between the two flanking transcripts of the adjacent genes in the UCSC annotation table.

### Statistical analysis

#### Mixture model

Because of the substantial existence of genes without any *Alus*, a mixture model was proposed to characterize the distribution of gene level intron *Alu *density within each chromosome or the cancer gene class. It consists of a Bernoulli probability mass function and a Gamma probability density function [34]. Let **x **= {*x_i_*} represent the intron *Alu *densities and *p_0 _*indicates the ratio of genes without *Alus*, the mixture model can be expressed as follows.(2)

In the implementation, we estimated *p_0 _*with *r*, the observed ratio of genes without *Alus*, and approximated the model as(3)

The model parameters θ (shape) and κ (scale) were estimated using maximum likelihood method (ML) implemented in the R package MASS [53]. The fitted and empirical distributions were compared using Q-Q plots.

#### Two-step regression analysis

This method was specially developed to analyze the gene-level effect of 5'-TTAAAA motif on the integration of *Alu *elements. The motivation is that a single linear model is not sufficient to analyze the observed data where a substantial proportion (e. g. approximately 30% in human genome) of genes contains no *Alu *elements and, as a result, we cannot conduct the logarithm transformation of *Alu *densities to resemble a normal distribution. The proposed method consists of a logistic regression model and a simple linear model. Below are the mathematical expressions of these two models.(4)

In Model-1, *z _i _*∈ {1,0} indicates if gene *i *has at least one *Alu *or no *Alus* in the intron region(s). *x_i _*is the intron motif density, and *l_i _*is the log10 transformed adjusted sequence length (with *Alu *sequences excluded from the calculation). In Model-2, for a specific gene j, *x_j_, y_j_*, *l_j_*, and *e_j _*are the motif density, *Alu *density, log10 adjusted sequence length of this gene, and random noise, respectively. (*μ*,*α*, *β*) and (μ*,α*, β*) are the parameter sets of the two models. Model-1 tests the effect of the motif density on the presence or absence of *Alu *elements in intron regions for all multi-exon genes. Model-2 tests the effect of the motif density on the intron *Alu *density for the genes with at least one *Alu *in the intron region(s). We conducted the logistic regression analysis using the procedure *lrm *included in the R package "Design". The pseudo contribution rate of the intron motif to the total variability was measured as the increase of Nagelkerke R^2 ^index [54] due to adding the density (x) to the reduced model which contained *l *as the only explainable variable. The simple regression analysis was conducted with the procedure *lm *in the R package "stats" and the contribution rate of intron motif to the total variability was measured as the increase of statistic R^2 ^due to adding the density (x) to the reduced model. The multi-testing across chromosomes was addressed by BH method [32]. It is worth noting that in both models, gene size was included as an independent variable. This is because our preliminary study showed that gene size had a significant effect on the presence or absence of *Alu *in intron region(s) for most chromosomes.

#### The association of Alu integration and mutation types of cancer genes

The effect of *Alu *density on mutation feature based classification (a binary variable) was analyzed using a logistic regression model. The formula was similar to the Model-1 in equation (4) with the binary variable *z_i _*indicating the category of the cancer gene *i*. For example, the value of *z_i _*is 1 if the mutation type of gene *i *is "recessive", or 0 if the mutation is "dominant". When comparing a specific gene class with the entire gene set in the genome, we assigned 1 or 0 to *z_i _*depending on gene *i *within the studied class or not.

#### Clustering analysis

Agglomerative hierarchical clustering algorithm was used to group the multi-exon cancer genes. The used features included the intron *Alu *density, the exon *Alu *density, the density of intron *Alu *pairs, the number of *Alu*-exon-*Alu *triplets, and GC content (*Alu *sequences were excluded from the calculation). The algorithm was executed with complete linkage and Euclidean distance as the parameters. The dendrogram was cut in a heuristic way.

## Authors' contributions

WZ carried out the statistical analysis and drafted the manuscript. AE, WF, PD and KZ helped with the experimental design, provided editorial comments and participated in writing. PD provided the biological interpretation. KZ supervised and coordinated the project. All authors read and approved the final manuscript.

## Supplementary Material

Additional file 1 **The Q-Q plots for fitting a Gamma distribution to the gene-level intron *Alu *densities in 20 chromosomes**. Click here for file

Additional file 2 **The analysis of genes deviating from the diagonal lines of the Q-Q plots for chromosomes-1, -3, -6, -13, -21, and -X**. Click here for file

Additional file 3 **The frequency distributions of intron and exon *Alu *densities of the cancer genes with somatic and germline mutations**. Click here for file

Additional file 4 **The frequency distributions of intron and exon *Alu *densities of the cancer genes with translocation and non-translocation mutations**. Click here for file

Additional file 5 **The distributions of 351 cancer genes measured by the *Alu*-related genomic features and GC content**. Click here for file

Additional file 6 **The Functional enrichment analysis of 5578 multi-exons genes without intron *Alus***. Click here for file
