# Supplementary material for: Alu distribution and mutation types of cancer genes
Source: BMC Genomics. 2011 Mar 23;12:157. doi: 10.1186/1471-2164-12-157 (PMC3074553; doi:10.1186/1471-2164-12-157)
Supplement: Additional file 1 — The Q-Q plots for fitting a Gamma distribution to the gene-level intron Alu densities in 20 chromosomes. [file 1471-2164-12-157-S1.PDF]

Additional File 1: The Q-Q plots for fitting a Gamma distribution to the gene-level intron *Alu* densities in 20 chromosomes. The dots represent the genes.

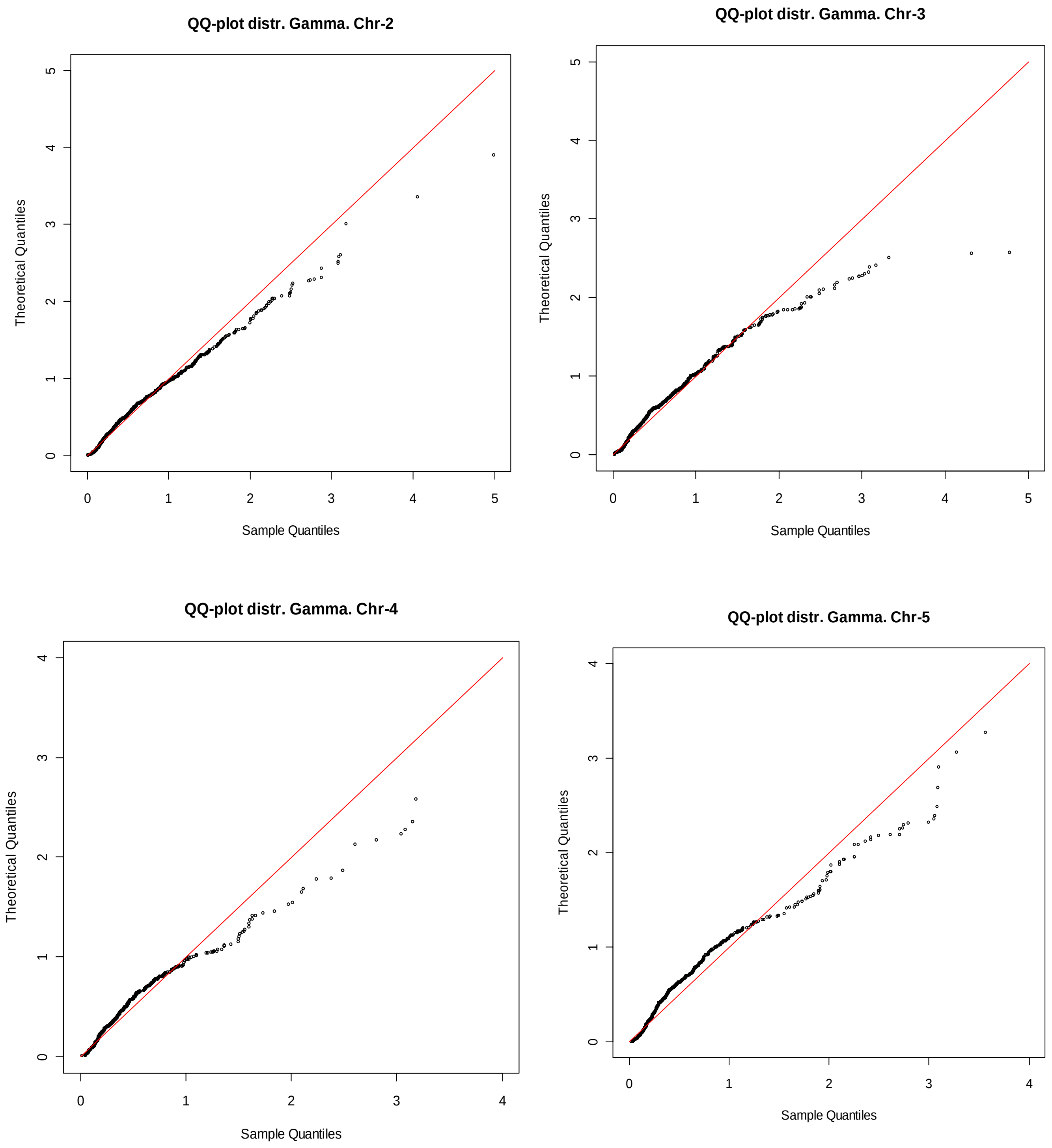

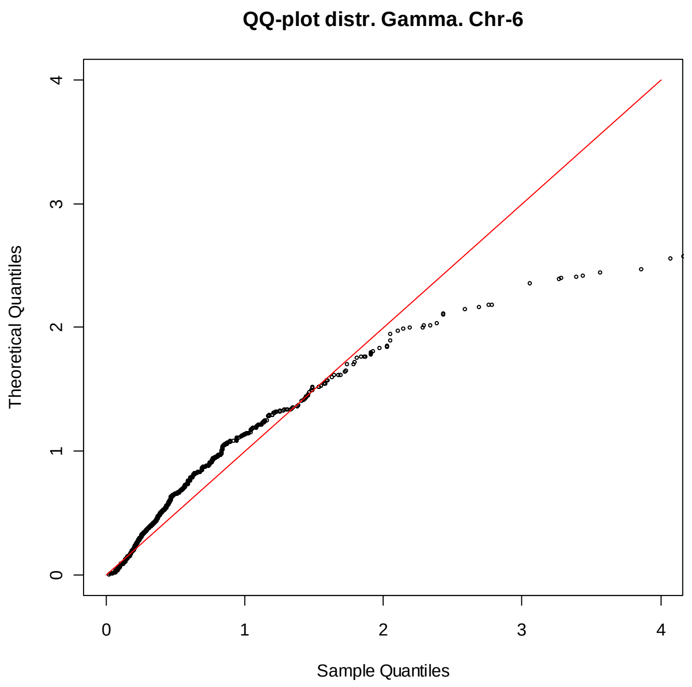
QQ-plot distr. Gamma. Chr-6
Theoretical Quantiles
Sample Quantiles
0
1
2
3
4

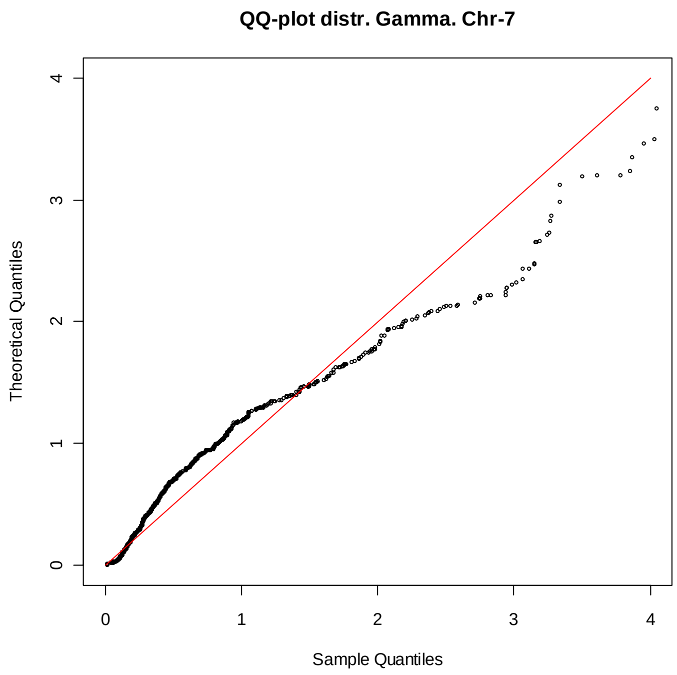
QQ-plot distr. Gamma. Chr-7
Theoretical Quantiles
Sample Quantiles
0
1
2
3
4

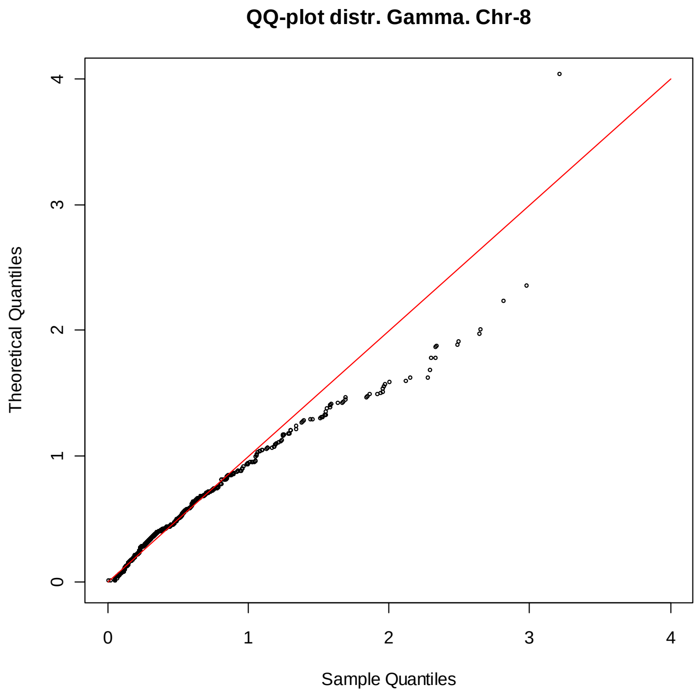
QQ-plot distr. Gamma. Chr-8
Theoretical Quantiles
Sample Quantiles
0
1
2
3
4

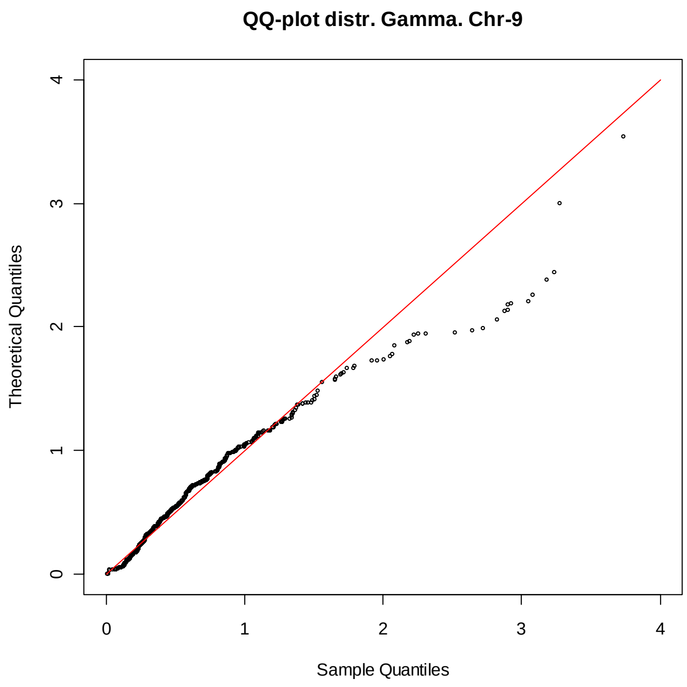
QQ-plot distr. Gamma. Chr-9
Theoretical Quantiles
Sample Quantiles
0
1
2
3
4

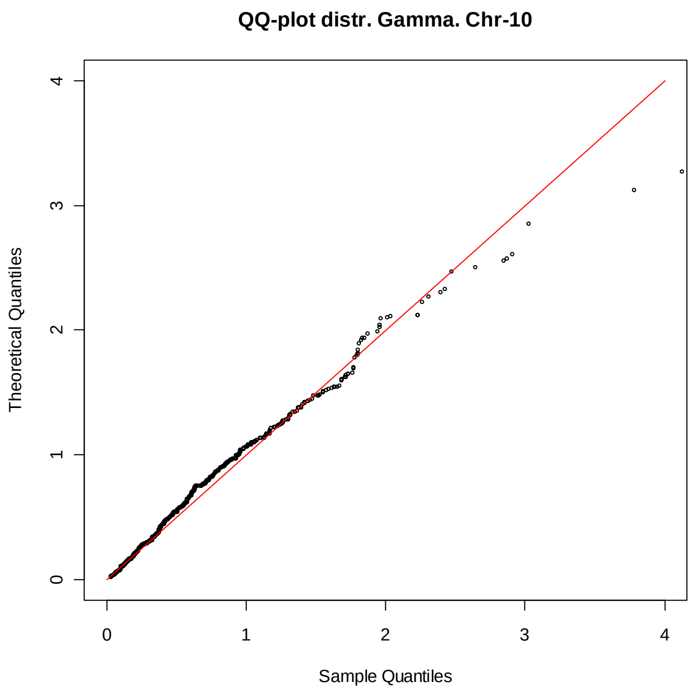
QQ-plot distr. Gamma. Chr-10
Theoretical Quantiles
Sample Quantiles

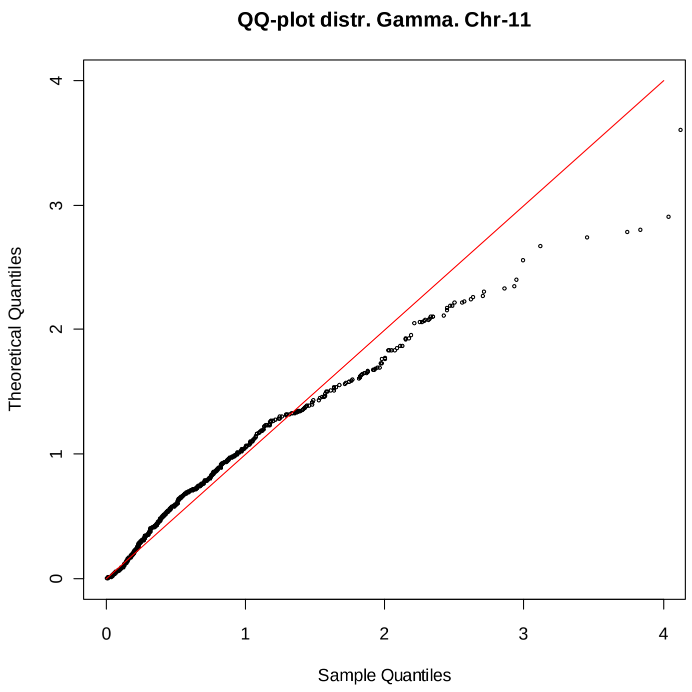
QQ-plot distr. Gamma. Chr-11
Theoretical Quantiles
Sample Quantiles

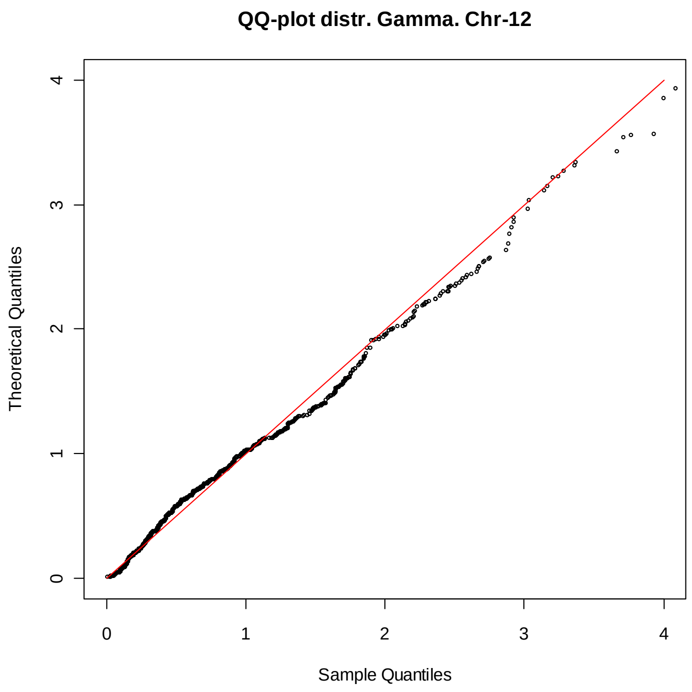
QQ-plot distr. Gamma. Chr-12
Theoretical Quantiles
Sample Quantiles

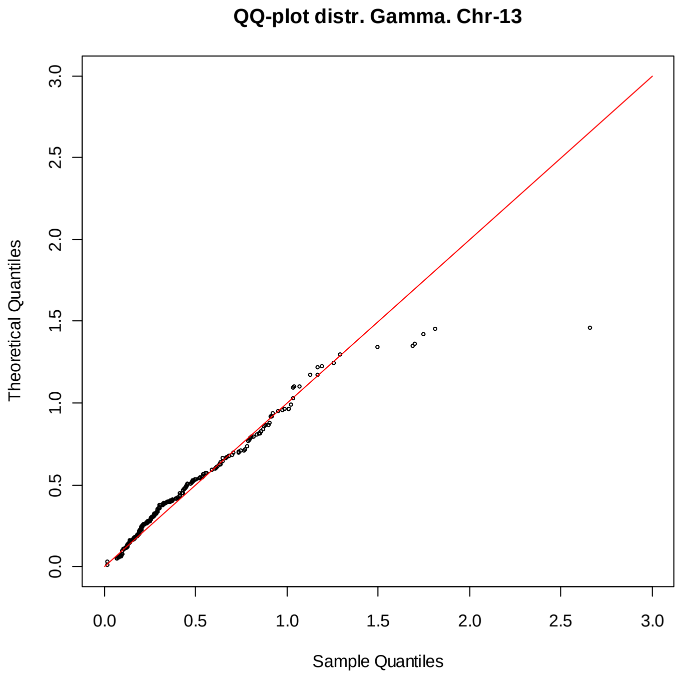
QQ-plot distr. Gamma. Chr-13
Theoretical Quantiles
Sample Quantiles

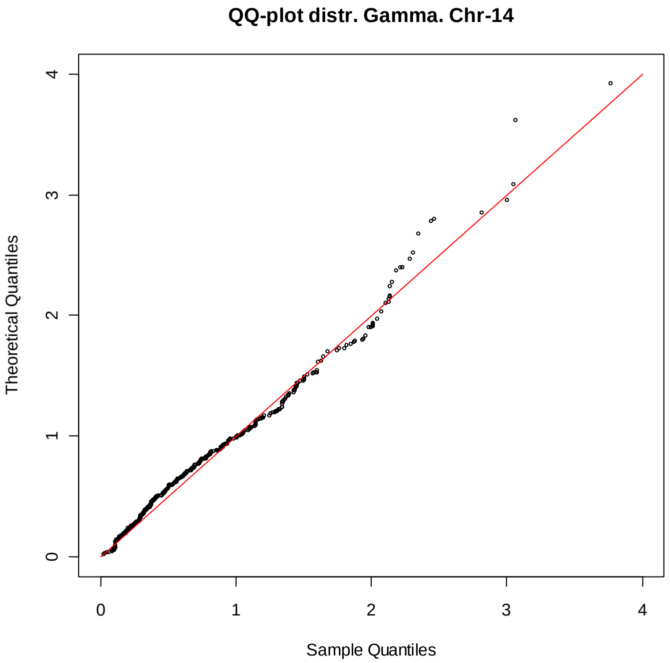
QQ-plot distr. Gamma. Chr-14
Theoretical Quantiles
Sample Quantiles

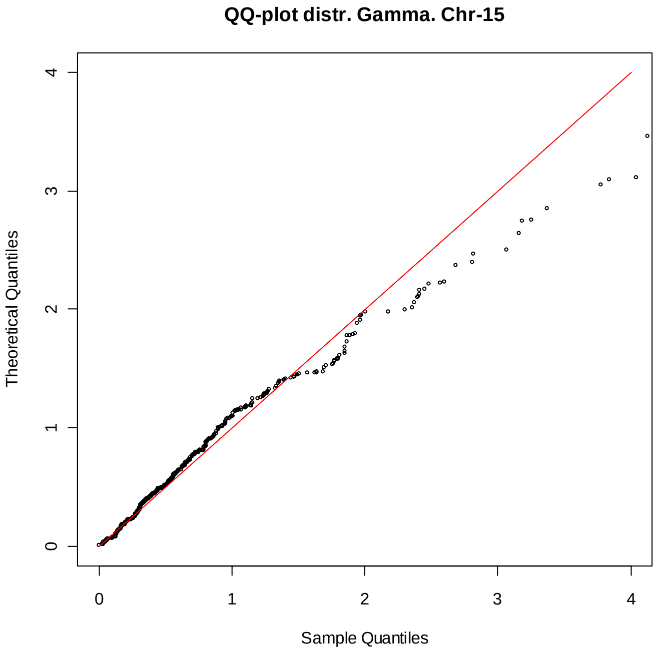
QQ-plot distr. Gamma. Chr-15
Theoretical Quantiles
Sample Quantiles

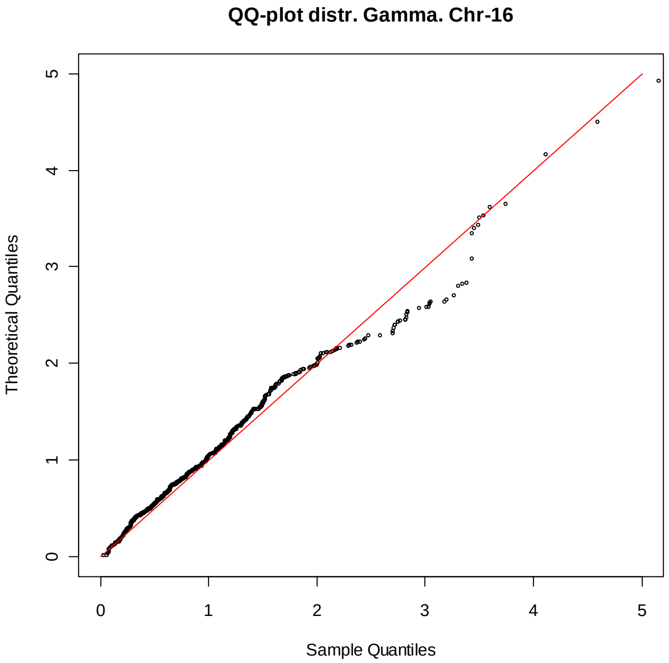
QQ-plot distr. Gamma. Chr-16
Theoretical Quantiles
Sample Quantiles

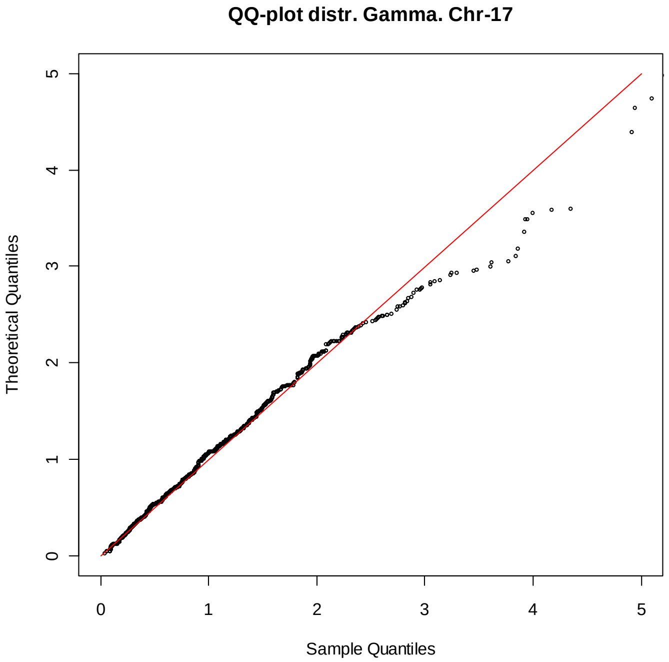
QQ-plot distr. Gamma. Chr-17
Theoretical Quantiles
Sample Quantiles

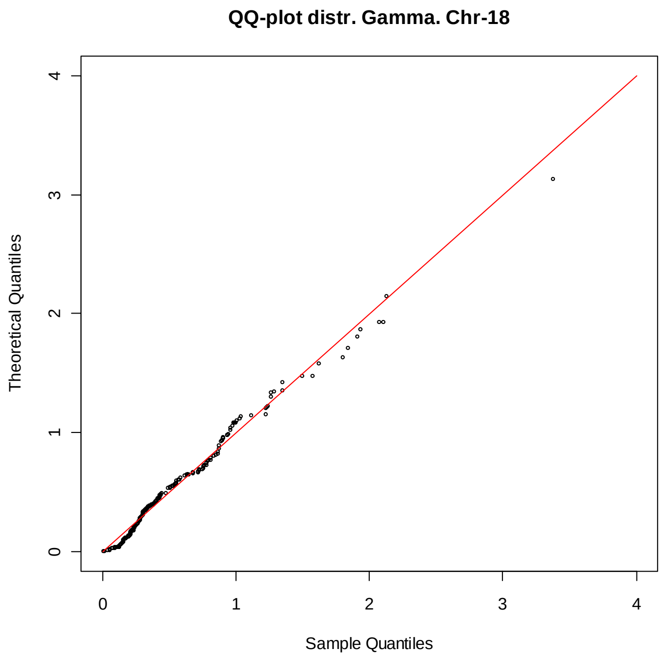
QQ-plot distr. Gamma. Chr-18
Theoretical Quantiles
Sample Quantiles
0
1
2
3
4

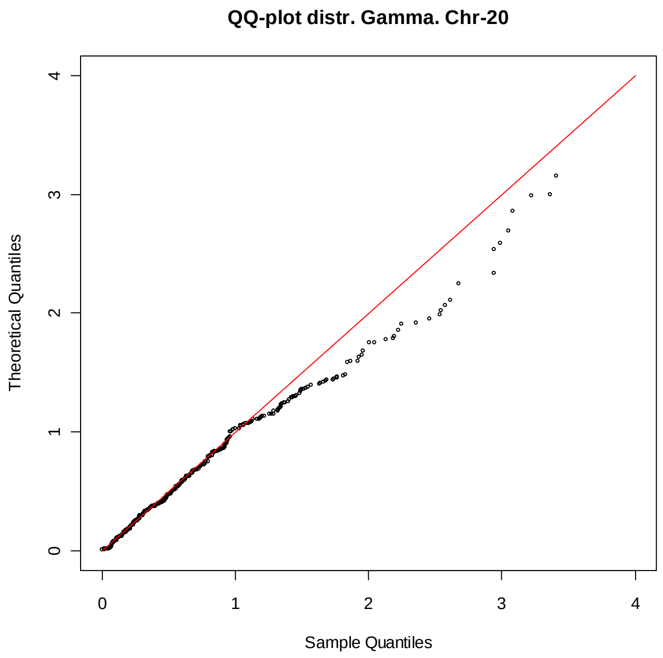
QQ-plot distr. Gamma. Chr-20
Theoretical Quantiles
Sample Quantiles
0
1
2
3
4

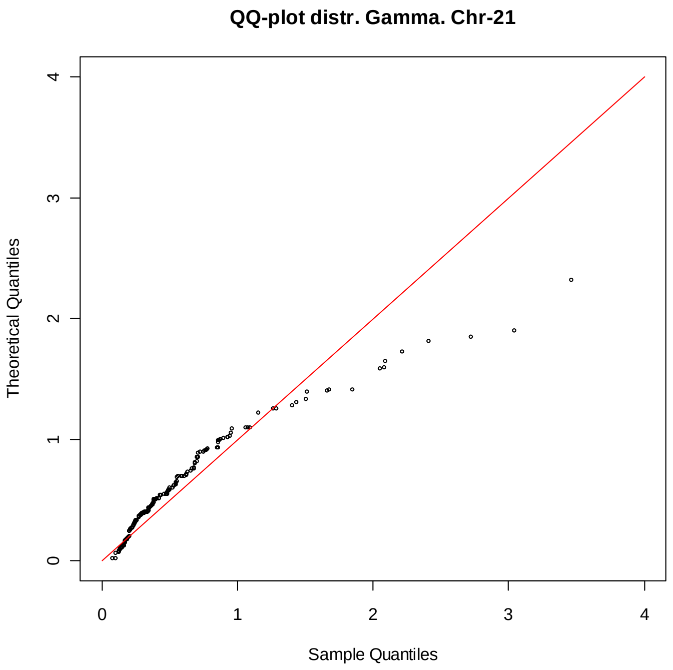
QQ-plot distr. Gamma. Chr-21
Theoretical Quantiles
Sample Quantiles
0
1
2
3
4

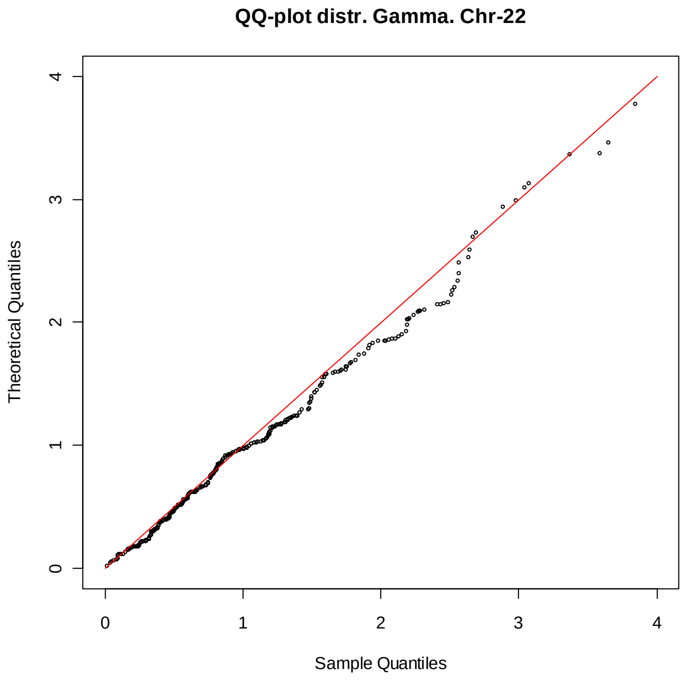
QQ-plot distr. Gamma. Chr-22
Theoretical Quantiles
Sample Quantiles
0
1
2
3
4
