# Supplementary material for: Alu distribution and mutation types of cancer genes
Source: BMC Genomics. 2011 Mar 23;12:157. doi: 10.1186/1471-2164-12-157 (PMC3074553; doi:10.1186/1471-2164-12-157)
Supplement: Additional file 2 — The analysis of genes deviating from the diagonal lines of the Q-Q plots for chromosomes-1, -3, -6, -13, -21, and -X. [file 1471-2164-12-157-S2.PDF]

Additional File 2: The analysis of genes deviating from the diagonal lines of the Q-Q plots for chromosomes-1, -3, -6, -13, -21, and -X

Symbol	Position	Size	Intron Alu density	Exon Alu density
C1orf189	1q21.3	6962	5.703	0.000
TRNAU1AP	1p35.3	25529	5.601	0.516
RBP7	1p36.22	18823	5.486	1.529
GABPB2	1q21.2	47928	5.343	0.512
GJA9	1p34	7551	4.895	0.000
LZIC	1p36.22	13051	4.732	0.000
GMEB1	1p35	46143	4.511	0.000
TAF12	1p35	39994	4.367	0.000
NMNAT1	1p36.22	42071	4.224	0.000
TMEM82	1p36.13	5561	4.023	0.000
SNAPIN	1q22	3181	3.980	0.000
LCE5A	1q21.3	1334	3.846	0.000
C3orf45	3p21.31	9028	6.078	0.000
SLC25A20	3p21.31	42047	4.774	0.000
QRICH1	3p21.31	64363	4.319	0.000
ISY1	3q21.3	31795	3.323	0.000
RHOA	3p21.3	52948	3.175	0.521
USP4	3p21.3	62960	3.088	0.000
PRKAR2A	3p21.3-p21.2	97178	3.086	0.000
C3orf10	3p25.3	11542	3.032	0.850
TCTEX1D2	3q29	27068	3.005	1.490
MON1A	3p21.31	21143	2.971	0.000
VHL	3p25.3	10428	2.965	0.692
LSM2	6p21.3	9570	5.876	0.000
HSPA1L	6p21.3	5440	5.372	0.000
LY6G6F	6p21	3689	4.926	0.000
C6orf153	6p21.1	7953	4.815	0.000
NCR3	6p21.3	3972	4.754	0.000
GTF3C6	6q21	9327	4.165	0.000

NUP43	6q25.1	22232	4.071	1.040
TUBA3C	13q12.11	8019	2.665	0.000
SKA3	13q11	23007	1.815	0.000
TRIM13	13q14	21461	1.749	0.288
FLT3	13q12	97319	1.704	0.000
C13orf1	13q14.3	23784	1.691	0.653
NDUFV3	21q22.3	16396	3.465	0.718
CBR3	21q22.2	11598	3.043	0.000
C21orf57	21q22.3	8622	2.720	0.000
CHAF1B	21q22.2	31437	2.413	0.000
DOPEY2	21q22.2	129734	2.213	0.130
TMEM50B	21q22.1	30834	2.095	0.429
MORC3	21q22.13	56457	2.082	0.000
C21orf58	21q22.3	22739	2.054	0.674
DSCR6	21q22.2	13094	1.848	0.896
IFNGR2	21q22.1	34627	1.681	1.351
TMEM187	Xq28	10656	4.127	0.000
AKAP14	Xq24	19057	3.647	0.000
