# Supplementary material for: Alu distribution and mutation types of cancer genes
Source: BMC Genomics. 2011 Mar 23;12:157. doi: 10.1186/1471-2164-12-157 (PMC3074553; doi:10.1186/1471-2164-12-157)
Supplement: Additional file 4 — The frequency distributions of intron and exon Alu densities of the cancer genes with translocation and non-translocation mutations. [file 1471-2164-12-157-S4.PDF]

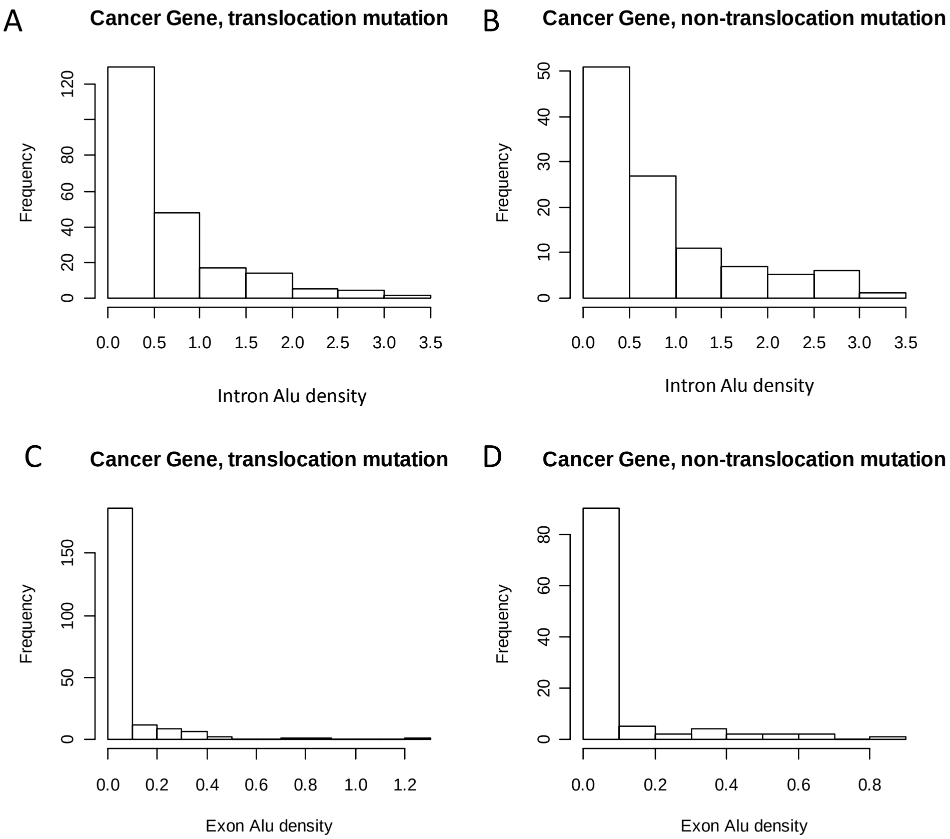

Additional File 4: The frequency distributions of intron and exon Alu densities of the cancer genes with translocation and non-translocation mutations.
