# Supplementary material for: Alu distribution and mutation types of cancer genes
Source: BMC Genomics. 2011 Mar 23;12:157. doi: 10.1186/1471-2164-12-157 (PMC3074553; doi:10.1186/1471-2164-12-157)
Supplement: Additional file 5 — The distributions of 351 cancer genes measured by the Alu-related genomic features and GC content. [file 1471-2164-12-157-S5.PDF]

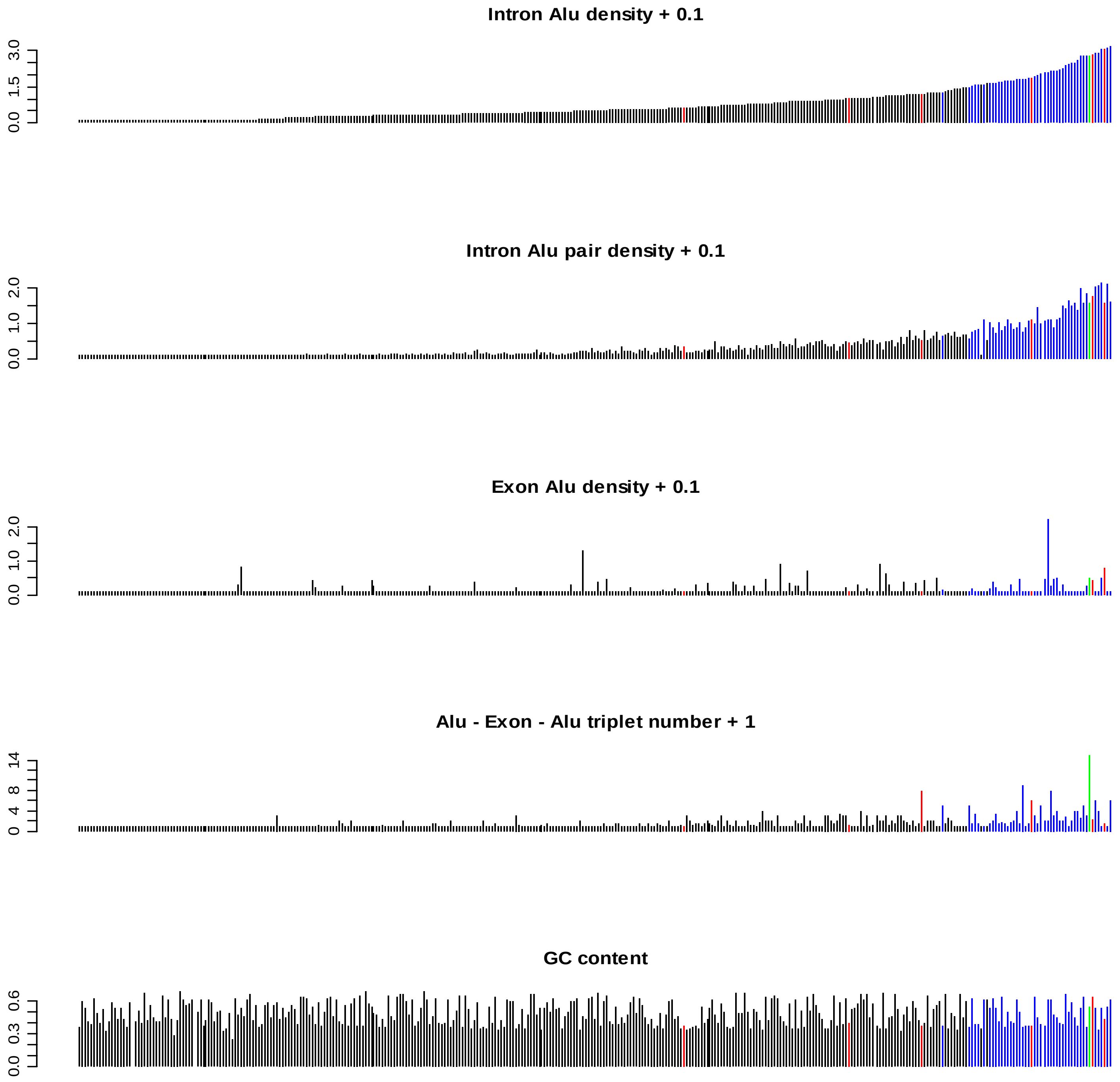

Additional File 5: The distributions of 351 cancer genes measured by the Alu-related genomic features and GC content. Each bar represents one gene and those genes are sorted in terms of the intron Alu density. The genes contained in CL1 and CL2 in **Figure 9** are marked with blue. Huntingtin interacting protein 1 gene (HIP1) that appears as a scalar in **Figure 9** is marked with green. The six genes (BRCA1, MLL, MSH2, VHL, MYB, and MLH1) in which Alu mediated recombination events were reported are marked with red. To ensure that all genes can be displayed on the plots, we increased the values of genes' Alu-related genomic features by a constant as indicated by the subtitles.
