# Supplementary material for: Alu distribution and mutation types of cancer genes
Source: BMC Genomics. 2011 Mar 23;12:157. doi: 10.1186/1471-2164-12-157 (PMC3074553; doi:10.1186/1471-2164-12-157)
Supplement: Additional file 6 — The Functional enrichment analysis of 5578 multi-exons genes without intron Alus. [file 1471-2164-12-157-S6.PDF]

Additional File 6: The Functional enrichment analysis of 5578 multi-exons genes without intron *Alus*

Category	Term	Count	%	adj.p
BP_5	GO:0010556~regulation of macromolecule biosynthetic process	772	14.424514	3.37E-15
BP_5	GO:0031326~regulation of cellular biosynthetic process	799	14.928999	1.75E-15
BP_5	GO:0006355~regulation of transcription, DNA-dependent	520	9.715994	1.91E-15
BP_5	GO:0045449~regulation of transcription	717	13.396861	1.74E-15
BP_5	GO:0051252~regulation of RNA metabolic process	529	9.8841555	1.66E-15
BP_5	GO:0019219~regulation of nucleobase, nucleoside, nucleotide…	764	14.275037	3.56E-15
BP_5	GO:0006414~translational elongation	62	1.1584454	4.39E-15
BP_5	GO:0009952~anterior/posterior pattern formation	76	1.4200299	9.76E-15
CC_5	GO:0022626~cytosolic ribosome	53	0.990284	6.25E-14
BP_5	GO:0010468~regulation of gene expression	768	14.349776	1.00E-13
BP_5	GO:0009887~organ morphogenesis	200	3.7369208	9.02E-13
BP_5	GO:0009888~tissue development	217	4.054559	8.20E-10
CC_5	GO:0044445~cytosolic part	71	1.3266069	2.39E-09
CC_5	GO:0033279~ribosomal subunit	63	1.17713	1.68E-09
CC_5	GO:0005840~ribosome	89	1.6629297	7.71E-09
MF_5	GO:0008009~chemokine activity	30	0.5605381	8.87E-08
BP_5	GO:0060429~epithelium development	89	1.6629297	2.54E-07
MF_5	GO:0042379~chemokine receptor binding	30	0.5605381	3.82E-07
CC_5	GO:0022627~cytosolic small ribosomal subunit	27	0.5044843	3.73E-07
BP_5	GO:0007398~ectoderm development	78	1.4573991	2.81E-06
BP_5	GO:0008544~epidermis development	73	1.3639761	4.46E-06
BP_5	GO:0031424~keratinization	27	0.5044843	4.97E-06
BP_5	GO:0030855~epithelial cell differentiation	58	1.083707	9.36E-06
BP_5	GO:0007417~central nervous system development	138	2.5784753	9.55E-06
BP_5	GO:0042742~defense response to bacterium	50	0.9342302	1.08E-05
BP_5	GO:0048663~neuron fate commitment	26	0.4857997	1.13E-05
CC_5	GO:0022625~cytosolic large ribosomal subunit	24	0.4484305	1.75E-05
BP_5	GO:0048706~embryonic skeletal system development	37	0.6913303	7.26E-05
BP_5	GO:0048562~embryonic organ morphogenesis	54	1.0089686	1.06E-04
CC_5	GO:0015935~small ribosomal subunit	32	0.5979073	7.25E-05
BP_5	GO:0048699~generation of neurons	167	3.1203288	1.32E-04
BP_5	GO:0022008~neurogenesis	177	3.3071749	1.60E-04
BP_5	GO:0031328~positive regulation of cellular biosynthetic process	197	3.680867	2.16E-04
BP_5	GO:0007423~sensory organ development	80	1.4947683	2.43E-04
BP_5	GO:0048568~embryonic organ development	64	1.1958146	2.62E-04
MF_5	GO:0005184~neuropeptide hormone activity	16	0.2989537	4.32E-04
BP_5	GO:0009891~positive regulation of biosynthetic process	198	3.6995516	3.50E-04
BP_5	GO:0007420~brain development	95	1.7750374	4.52E-04
CC_5	GO:0015934~large ribosomal subunit	32	0.5979073	3.32E-04

BP_5	GO:0006412~translation	106	1.980568	4.43E-04
BP_5	GO:0009913~epidermal cell differentiation	33	0.6165919	7.80E-04
BP_5	GO:0001708~cell fate specification	26	0.4857997	8.89E-04
BP_5	GO:0051173~positive regulation of nitrogen compound metabolic process	183	3.4192825	8.66E-04
BP_5	GO:0045935~positive regulation of nucleobase, nucleoside, nucleotide …	178	3.3258595	8.91E-04
BP_5	GO:0045941~positive regulation of transcription	163	3.0455904	9.59E-04
BP_5	GO:0010557~positive regulation of macromolecule biosynthetic process	185	3.4566517	9.56E-04
BP_5	GO:0010628~positive regulation of gene expression	167	3.1203288	9.64E-04
CC_5	GO:0001533~cornified envelope	15	0.2802691	0.0013903
BP_5	GO:0045893~positive regulation of transcription, DNA-dependent	140	2.6158445	0.0016812
BP_5	GO:0048665~neuron fate specification	12	0.2242152	0.0016427
BP_5	GO:0051253~negative regulation of RNA metabolic process	111	2.073991	0.0016817
BP_5	GO:0048705~skeletal system morphogenesis	44	0.8221226	0.0017461
BP_5	GO:0021871~forebrain regionalization	10	0.186846	0.0021114
BP_5	GO:0048704~embryonic skeletal system morphogenesis	27	0.5044843	0.0021972
BP_5	GO:0051254~positive regulation of RNA metabolic process	140	2.6158445	0.0022759
CC_5	GO:0042612~MHC class I protein complex	17	0.3176383	0.0021874
BP_5	GO:0045892~negative regulation of transcription, DNA-dependent	108	2.0179372	0.0031567
MF_5	GO:0046914~transition metal ion binding	624	11.659193	0.0081091
CC_5	GO:0005833~hemoglobin complex	10	0.186846	0.0054812
BP_5	GO:0048729~tissue morphogenesis	61	1.1397608	0.0065572
BP_5	GO:0043009~chordate embryonic development	100	1.8684604	0.0069502
BP_5	GO:0001709~cell fate determination	18	0.3363229	0.007023
BP_5	GO:0021510~spinal cord development	20	0.3736921	0.0073179
BP_5	GO:0048754~branching morphogenesis of a tube	28	0.5231689	0.0096395
